# The Human Kinome Targeted by FDA Approved Multi-Target Drugs and Combination Products: A Comparative Study from the Drug-Target Interaction Network Perspective

**DOI:** 10.1371/journal.pone.0165737

**Published:** 2016-11-09

**Authors:** Ying Hong Li, Pan Pan Wang, Xiao Xu Li, Chun Yan Yu, Hong Yang, Jin Zhou, Wei Wei Xue, Jun Tan, Feng Zhu

**Affiliations:** 1 Innovative Drug Research and Bioinformatics Group, Innovative Drug Research Centre and School of Pharmaceutical Sciences, Chongqing University, Chongqing, China; 2 Institute of Bioinformation, Chongqing University of Posts and Telecommunications, Chongqing, China; Tianjin University, CHINA

## Abstract

The human kinome is one of the most productive classes of drug target, and there is emerging necessity for treating complex diseases by means of polypharmacology (multi-target drugs and combination products). However, the advantages of the multi-target drugs and the combination products are still under debate. A comparative analysis between FDA approved multi-target drugs and combination products, targeting the human kinome, was conducted by mapping targets onto the phylogenetic tree of the human kinome. The approach of network medicine illustrating the drug-target interactions was applied to identify popular targets of multi-target drugs and combination products. As identified, the multi-target drugs tended to inhibit target pairs in the human kinome, especially the receptor tyrosine kinase family, while the combination products were able to against targets of distant homology relationship. This finding asked for choosing the combination products as a better solution for designing drugs aiming at targets of distant homology relationship. Moreover, sub-networks of drug-target interactions in specific disease were generated, and mechanisms shared by multi-target drugs and combination products were identified. In conclusion, this study performed an analysis between approved multi-target drugs and combination products against the human kinome, which could assist the discovery of next generation polypharmacology.

## Introduction

**Fig 1:**
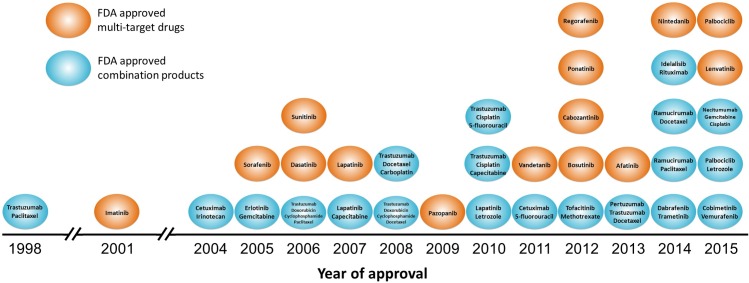
The approval timeline of FDA approved multi-target drugs and combination products against the human kinome.

Intensive efforts in exploring novel targets from the genome of human and other infectious species [1–3] have discovered hundreds of successful (targeted by approved drugs), hundreds of clinical trial (targeted by clinical trial drugs), and thousands of research targets (targeted by investigational agents only) [4]. Over the past two decades, kinases have become one of the most intensively studied protein classes in the target and drug discovery, with 46 drugs approved by the U.S. Food and Drug Administration (FDA) [4–6]. However, only a small portion (< 10%) of the human kinome has been established to yield successful targets [5,7]. Distinct from the traditional “one drug one target” philosophy, the polypharmacology refers to a novel paradigm by modulating more than one etiological target [8]. The concept of polypharmacology involves drugs against several targets of disease-related pathways [9] and combination products acting on distinct targets of various physiological responses [10,11]. Among those 46 FDA approved drugs targeting the human kinome, 15 are multi-target drugs, and 14 are approved for use in combination with other drugs [12]. Based on our review of FDA official website [12], imatinib is the first approved multi-target kinase inhibitor, and trastuzumab in combination with paclitaxel are the first approved combination product targeting the human kinome. As shown in **Fig 1**, a total of 6 multi-target drugs and 7 combination products are approved before 2010, and there is a steady growth after 2010 to 9 multi-target drugs and 13 combination products. Due to the emerging necessities for treating complex diseases by the means of polypharmacology, it is of great interests to identify novel efficacious target pairs from the human kinome [13–15].

The approved multi-target kinase inhibitors cope with the multifactorial nature of complex diseases by simultaneously aiming at multiple targets [16–20]. In the meantime, the approved combination products targeting the human kinome produce synergistic effects triggered by actions converging at a specific pathway site, which enhances its clinical capacity for treating diseases of great complexity [10,21,22]. So far, several studies have been conducted to assess the applicability domain of both multi-target drug and combination product and to analyze their advantages over another [23,24]. The combination products can reduce unwanted compensatory mechanisms [24], achieve high efficacy and selectivity [10], and prevent the drug resistance [25]. However, their effectiveness can be attenuated by the pharmacokinetic variations among individual ingredients and the problem of drug-drug interaction [26]. The multi-target drugs provide an effective approach to avoid the problem of drug–drug interactions [27], but it is very difficult to design an efficacious multi-target drug since the design of a single target drug has already been problematic [23]. So far, the differential advantages of the multi-target drugs and the combination products are still under debate. Therefore, it is in urgent need to conduct a comprehensive comparative study between the multi-target drugs and the combination products.

In this study, the multi-target drugs and the combination products targeting the human kinome approved by FDA were compared. Firstly, their drug-target interaction networks were drawn and analyzed [28], and corresponding targets of great popularity were identified. So far, several works based on the analysis of biological networks have been successfully conducted for prioritizing disease-related microRNA [29], inferring microRNA-disease associations [30,31], and so on. Secondly, the biochemical classes of their aimed target pairs were discussed, and the preferred combinations of biochemical class were discovered. Finally, diseases treated by both multi-target drugs and combination products were identified. Popular efficacious targets of the same disease were also explored. In sum, this study provided a comprehensive comparative analysis between FDA approved multi-target drugs and combination products against the human kinome from the drug-target interaction network perspective, which could help to identify novel target pairs efficacious for the next generation polypharmacology.

## Materials and Methods

**Fig 2:**
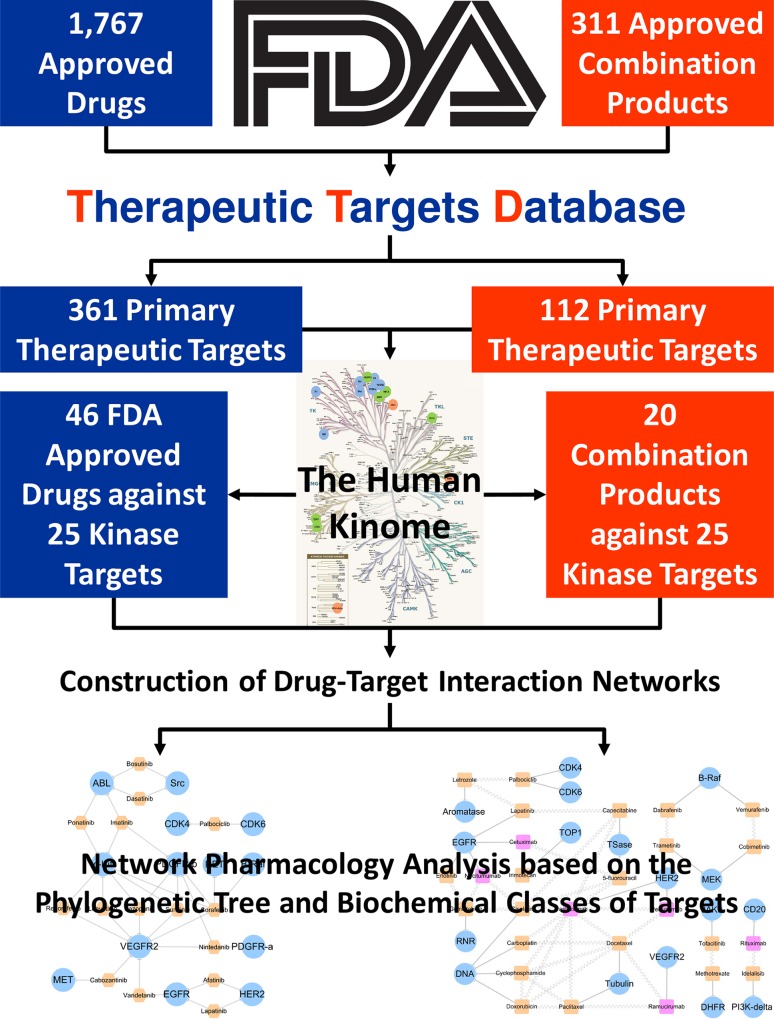
A framework of proposed comparative strategy used in this study.

The proposed strategy used in this study to compare the human kinome targeted by multi-target drugs and combination products from the drug-target interaction network perspective was illustrated in **Fig 2**. In general, this comparative study could be separated into five steps: 1) collection of approved drugs and combination products from FDA official website; 2) identification of the primary therapeutic target of drugs from the therapeutic targets database; 3) mapping the successful targets to the human kinome; 4) construction of drug-target interaction networks; and 5) network pharmacology analysis based on the phylogenetic tree and biochemical classes of targets.

### Data collection

All FDA approved multi-target drugs against the human kinome were collected from the FDA website (Drugs@FDA, http://www.accessdata.fda.gov/scripts/cder/drugsatfda/) by following steps. Firstly, 1,767 FDA approved drugs were identified and collected from Drugs@FDA together with their aimed diseases. Secondly, based on the established criterion of primary therapeutic targets provide in **S1 Table**, the primary therapeutic targets of these drugs were matched from the *Therapeutic Target Database* (TTD, http://database.idrb.cqu.edu.cn/TTD/) [4], and in total 1,521 approved drugs against 361 primary therapeutic targets were identified. Thirdly, the biochemical classes of protein targets were discovered by mapping their protein name to the *UniProt database* [32,33]. Drugs against at least one kinase target (EC: 2.7) were identified, which included 46 drugs aiming at 25 targets. Finally, drugs with more than one target were defined as multi-target drugs, and were further confirmed by comprehensive literature search. In total, 15 multi-target drugs against 13 targets in the human kinome were shown in **S2 Table**.

Combination products approved by FDA against at least one kinase target were also collected from the FDA official website. Firstly, more than 300 combinations were identified, and their disease indications were collected. Secondly, targets of combination products were matched from the TTD [4], and 264 approved combination products against 112 therapeutic targets were identified. Thirdly, the biochemical class of each target was collected from the *UniProt database* [32,33]. Only the combinations against at least one target of the kinase class were analyzed in this study, which included 20 combination products against 17 targets (**S3 Table**). 14 targets were from enzyme family with 9 belonging to the kinase class (EC: 2.7), while the remaining 3 were in the families of antigen, structural protein and nucleic sequence.

### Identification of the primary therapeutic target of the FDA approved drugs

Primary therapeutic targets of approved multi-target drugs and combination products were identified by a target validation process, which requires three steps of determination [34,35]. 1) whether the aimed target was expressed in the disease-relevant cells or tissues? 2) whether the intended target could be effectively modulated by a drug or drug-like molecule with adequate activity in biochemical assay? 3) whether modulation of target in cell or animal models ameliorated the relevant disease phenotype? In this study, literature search of target validation data in PubMed database [36] was conducted, and three types of validation data were collected, which include experimentally determined potency of drugs against their primary target, observed potency or effects of drugs against disease models linked to their primary target, and the observed effects of target knockout, knockdown, transgenetic, RNA interference, antibody and antisense of the *in-vivo* model. Only the targets with full target validation information were defined as primary one of the corresponding drug.

### Mapping the successful targets to the human kinome

**Fig 3:**
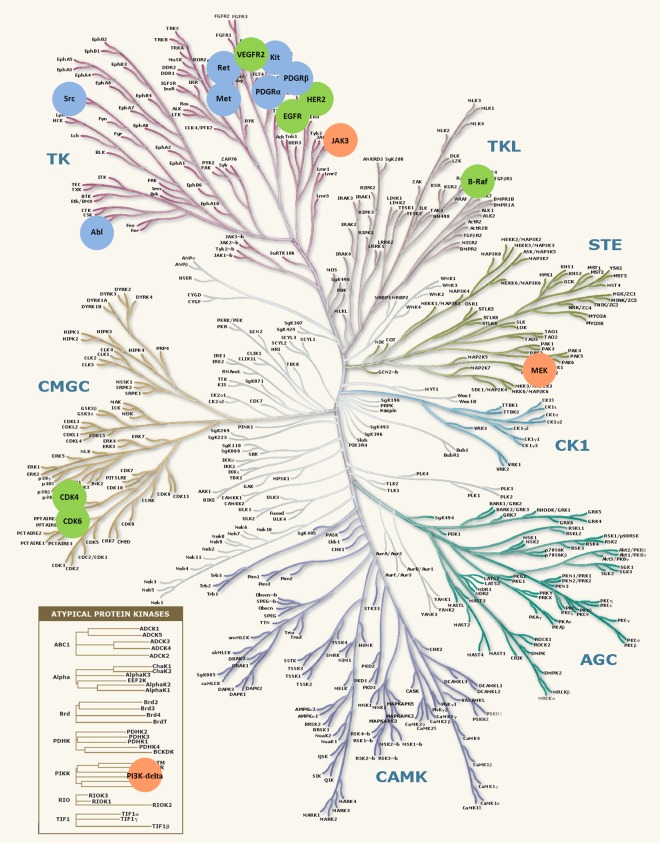
Targets of approved multi-target drugs and combination products in the phylogenetic tree of the human kinome adapted from Manning and colleagues [7]. The targets of both multi-target drugs and combination products were highlighted by green circle, while the targets of either multi-target drugs or combination products were highlighted by blue and orange circle respectively.

The human kinome containing 556 kinase genes could be represented by a phylogenetic tree [7]. In order to map all successful targets of the studied multi-target drugs and combination products to the tree of human kinome, the gene names of each successful target were identified from the *UniProt database* [32,33]. As shown in **Fig 3**, the targets of both multi-target drugs and combination products were highlighted by green circle, while the targets of either multi-target drugs or combination products were highlighted by blue and orange circle respectively.

### Construction of drug-target interaction network

**Fig 4:**
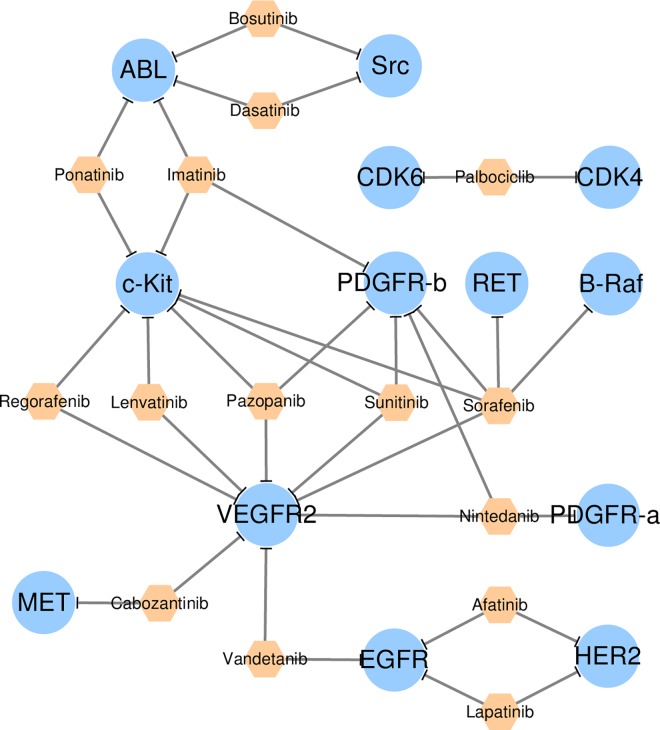
The drug-target interaction network of the FDA approved multi-target drugs. Drugs were represented by orange hexagon, and targets were shown by blue ellipse. Drug-target interactions were displayed by edges with shapes of arrow for activation and “T” for inhibition.

**Fig 5:**
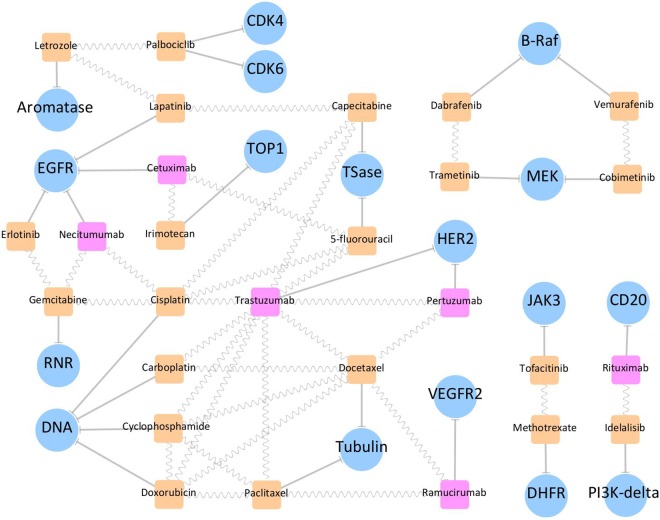
The drug-target interaction network of the FDA approved combination products. Small molecular drugs in a specific combination product were represented by round rectangle in orange, and monoclonal antibodies were shown by round rectangle in magenta. The combination products were shown by round rectangles connected by sine-wave, and targets were shown by blue ellipse. Drug-target interactions were displayed by edges with shapes of arrow for activation and “T” for inhibition.

The drug-target interaction networks were constructed and displayed using the *Cytoscape* [37], which was a stand-alone platform for visualizing molecular interactions. 15 multi-target drugs together with their corresponding 13 targets were uploaded to and displayed in the *Cytoscape*. As shown in **Fig 4**, multi-target drugs were represented by orange hexagon, and all targets were shown by blue ellipse. In the meantime, 20 combination drugs along with their aiming 17 targets were inputted and shown in the *Cytoscape*. The network of drug-target interaction was also provided in **Fig 5** with the representation of target the same as that in **Fig 4**. Small molecular drugs in a specific combination product were represented by round rectangle in orange, while the monoclonal antibodies were shown in magenta. The combination products were shown by round rectangles connected by sine-wave. Drug-target interactions were displayed by edges with shapes of arrow for activation and “T” for inhibition.

### Construction of phylogenetic tree of targets aimed by FDA approved drugs

**Fig 6:**
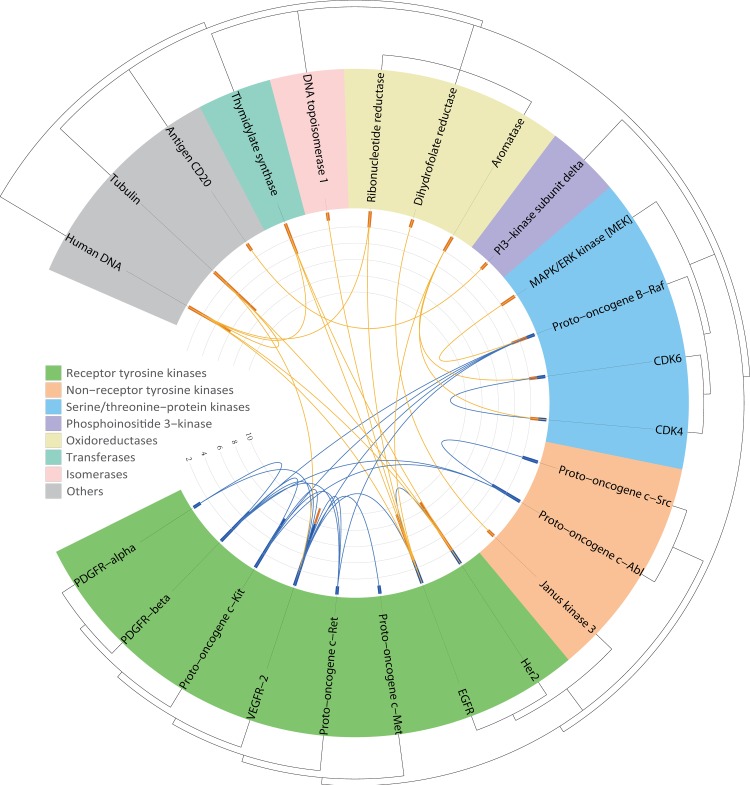
Phylogenetic tree representing the homology distance among all targets targeted by approved multi-target drugs and combination products. The connections shown in the center represented the target pairs aimed by the multi-target drugs (lines in blue) and the combination products (lines in orange). Bars at the end of each connecting line defined the number of multi-target drugs (blue bar) and combination products (orange bar).

Sequences of all targets aimed by any of those approved multi-target drugs and combination products were collected from the *UniProt database* [32,33], and a phylogenetic tree representing the homology distance among those targets was constructed as follows. Firstly, *HMMER* [38,39] was used to integrate the hidden Markov model for multiple sequence alignment of those studied protein targets. Secondly, the *FastTree 2*.*1*.*7* [40] was used to construct the approximately-maximum-likelihood phylogenetic tree based on the previous alignment results. For targets with distant homology hardly aligned by *HMMER*, their distance relationships were constructed based on the nomenclature for enzymes and protein family defined by the *Pfam database* [39]. Thirdly, the *iTOL3*.*0* [41] was applied to display the tree in **Fig 6**. The connections shown in the center of the figure represented the target pairs aimed by the multi-target drugs (lines in blue) and the combination products (lines in orange).

### Sub-network of drug-target interaction generated by disease classification

The World Health Organization (WHO) provided the International Classification of Diseases (ICD) as the standard diagnostic tool for epidemiology, health management and clinical purpose. By mapping diseases of all approved multi-target drugs and combination products in this study to the ICD code, sub-networks based on ICD could be generated. Firstly, the multi-target drugs and the combination products of the same disease class (level 2 of ICD-10) were identified, and their corresponding targets were collected. As a result, 11 sub-networks were identified. Secondly, each sub-network was displayed by the *Cytoscape* [37]. Among them, only 4 were treated by both multi-target drugs and combination products (**S1 Fig**). The definition of hexagon, ellipse and round rectangle was the same as that in the **Figs 4** and **5**. All multi-target drugs were highlighted by an orange hexagon line.

## Results and Discussion

### Successful targets in human kinome of the multi-target drugs and the combination products

As illustrated in **Fig 3**, only a very small portion (16 out of 556, ~2.9%) of the human kinome was identified as targeted by either the multi-target drugs or the combination products approved by FDA. There were 6 targets (VEGFR2, EGFR, HER2, CDK4, CDK6 and B-Raf) inhibited by both approved multi-target drugs and combination products. The biochemical classes of these 6 targets included the tyrosine kinase class (TK), the tyrosine kinase like class (TKL) and the CDK, MAPK, GSK3 and CLK kinase class (CMGC). In the meantime, 3 targets (JAK3, MEK and PI3K-delta) were aimed only by the combination products, the biochemical classes of which were distinct from each other (the TK class, the homologs of sterile kinase class (STE) and the PIKK class of protein kinases). Moreover, 7 targets (Src, Abl, Ret, Met, PDGFR-alpha, PDGFR-beta and Kit) inhibited only by the multi-target drugs were all in the TK class. Overall, the majority (10 out of 13) of the kinases targeted by the approved multi-target drugs and about half (4 out of 9) of the kinases inhibited by the approved combination products were from the TK class, which revealed it as the most productive class of target in the human kinome.

### Drug-target interaction networks of approved multi-target drugs and combination products

The network of the multi-target drugs and their corresponding targets were illustrated in **Fig 4**. As a frequently used statistical concept in network analysis, degree was applied to assess the interactions between targets and drugs. Degree of a specific node (drug or target) referred to the number of edges (interaction from other nodes) connected to this node. As shown in **Fig 4**, the maximum degree of the multi-target drugs was 5, and the minimum one was 2. In particular, 1, 4 and 10 multi-target drugs inhibited 5, 3 and 2 primary targets, respectively. Drug of the highest degree was sorafenib, reflecting the complex nature of its approved disease indications (hepatocellular carcinomas, renal cell carcinoma and differentiated thyroid carcinoma).

The maximum degree of targets was 8, and the minimum one was 1. Particularly, 1, 1, 1, 1, 1, 2 and 6 targets were targeted by 8, 7, 5, 4, 3, 2 and 1 multi-target drugs, respectively. Targets of high degree (>5) were VEGFR2 and c-Kit. VEGF and its receptor VEGFR2 were extensively tested primary therapeutic targets currently in clinical for renal cell carcinoma (RCC), and the development of RCC was proved to be relied on VEGF signaling and its receptor [42]. Similar to VEGFR2, c-Kit was proposed essential in the occurrence and development of the metastatic RCC, and its overexpression was well demonstrated in the chromophobe variety of RCC [43]. Thus, VEGFR2 and c-Kit were identified as the most popular therapeutic targets of approved multi-target drugs, and the binding of which could provide substantial influence on the pathogenesis of the RCC.

**Fig 5** illustrated the drug-target interaction of all FDA approved combination products together with their corresponding targets. The maximum degree of the combination products was 3, and the minimum one was 2. In particular, 6 and 14 combination products inhibited 3 and 2 primary therapeutic targets, respectively. The combination products of the highest degree (= 3) was used to treat 3 disease indications: 1) HER2-positive breast cancer (trastuzumab plus doxorubicin plus cyclophosphamide plus paclitaxel, trastuzumab plus docetaxel plus carboplatin, trastuzumab plus doxorubicin plus cyclophosphamide plus docetaxel), 2) HER2-positive metastatic gastric or gastroesophageal junction adenocarcinoma (5-FU plus trastuzumab plus cisplatin, trastuzumab plus cisplatin plus capecitabine), and 3) non-small cell lung cancer (necitumumab plus gemcitabine plus cisplatin). Several drugs (trastuzumab, cisplatin and doxorubicin) in those combinations were frequently appearing. Drugs in a combination could maximize their efficacy by synergistic effects, some of which were explicitly reported in previous study [10].

The maximum degree of targets was 4, and the minimum one was 1. Particularly, 2, 4 and 11 targets were aimed by 4, 2 and 1 combination drugs, respectively. Targets of the highest degree were EGFR and human DNA. EGFR enhanced aerobic glycolysis in the triple negative breast cancer (TNBC) to promote tumor growth and immune escape, and targeting its signaling could offer an applicable strategy to treat TNBC [44]. Moreover, cisplatin and doxorubicin were reported to form adduct with human DNA, which in turn resulted in DNA damage of the cancer cell [45]. Thus, EGFR and human DNA were discovered as popular therapeutic targets inhibited by FDA approved combination products.

### The phylogenetic tree and the biochemical classes of target pairs aimed by the multi-target drugs and the combination products approved by FDA

As shown in **Fig 6**, the phylogenetic tree representing the homology distance among targets aimed by those studied multi-target drugs and combination products was constructed. In total, 24 targets were shown. Among those targets, the majority (21 targets) were enzyme. In particular, 16, 3, 1 and 1 targets were from classes of kinase, oxidoreductase, transferase and isomerase, respectively. Clearly, the kinase class was the most popular target class for both multi-target drugs and combination products. 8, 4, 3 and 1 targets were from the family of receptor tyrosine kinase (in green), serine/threonine protein kinase (in blue), non-receptor tyrosine kinase (in earth yellow) and phosphoinositide 3-kinase (in magenta). In the center of **Fig 6**, lines connecting 2 targets represented target pairs aimed by the multi-target drugs (blue line) and the combination products (orange line), and significant differences could be observed. First of all, all target pairs of the multi-target drugs were within the kinase class with 11 pairs within the family of receptor tyrosine kinase. The rest of the 8 target pairs were within the non-receptor tyrosine kinase or between the receptor tyrosine kinase and (a) the non-receptor tyrosine kinase and (b) the serine/threonine protein kinase. In distinct contrast, target pairs of the combination products were not concentrated in kinase family, and only one target pair was within the family of serine/threonine protein kinase. The rest of the 16 target pairs were between kinase class and other classes such as transferase, structural protein, nucleic sequence, oxidoreductase, isomerase and antigen. In summary, the approved multi-target drugs tended to inhibit target pairs in the human kinome, especially the receptor tyrosine kinase family, while the approved combination products were able to target more diverse pair of targets with distant homology relationship. It is reasonable to conclude that if one wants to design drug to aim at targets of distant homology relationship, the best choice would be the combination product.

### Drug-target interaction sub-networks and popular targets of disease classes treated by both multi-target drugs and combination products

Based on the ICD classification, 4 disease classes treated by both multi-target drugs and the combination products were identified. Sub-networks of drug-target interaction within these 4 classes were displayed by the Cytoscape (**S1 Fig**). Definition of hexagon, ellipse and round rectangle was the same as that in **Figs 4** and **5**, and the multi-target drugs were highlighted by an orange hexagon line. Those 4 disease classes identified were breast cancer (C50-C50), cancer of respiratory and intrathoracic organs (C30-C39), cancer of digestive organs (C15-C26) and leukemia and lymphoma (C81-C96). All disease classes were in the super ICD class of “neoplasm”, which reflect the complex and multifactorial nature of cancer and the effectiveness of polypharmacology in the treatment of those 4 cancer types. As shown in **S1 Fig**, targets of some classes were inhibited by both multi-target drugs and combination products, while targets of other classes were aimed by either the multi-target drugs or the combination products.

#### Breast cancer

kinases targeted by both multi-target drugs and combination products for treating breast cancer included HER2, EGFR and CDK4/6. (1) the dual targeting of HER2 and EGFR with lapatinib prevented the subsequent activation of disease signaling in a group of high risk breast cancers [46,47]. As an anti-HER2 monoclonal antibody, trastuzumab was frequently in combination with other drugs, like cisplatin, to produce the synergistic effects via the “anti-counteractive” mechanism between the PI3k-AKT pathway and the cell cycle arrest mediated DNA repair [10]. (2) palbociclib blocked the progression of the cancer cell from G1 to S phase by targeting the cyclin D1-CDK4/6 complex [48,49], and letrozole decreased the estrogen effects through inhibiting the aromatase [50]. The combination of palbociclib and letrozole could therefore amplify their drug efficacy on ER-positive breast cancer by simultaneously blocking the cell cycle and the estrogen synthesis of cancer cells [50].

#### Leukemia and lymphoma

although no kinase was found to be targeted by both multi-target drugs and combination products for treating leukemia and lymphoma, popular targets of either the multi-target drugs or the combination products were identified. (1) popular targets of the multi-target drugs were ABL, Src and c-Kit. The mutation and translocation of ABL allowed the chronic myelogenous leukemia (CML) to progress [51–53]. Although the inhibition of the mutated ABL was efficacious to the CML, a substantial number of patients had failed to respond to the ABL inhibitors by developing drug resistance [52]. Src’s pathway [54] and c-kit’s mutation [55] were reported to contribute significantly to the drug resistance, and were the key adverse prognostic and predictive prognosis factors for leukemia [54,55]. Thus, the multi-target drugs inhibiting ABL together with Src or c-Kit were expected to counteract drug resistance of the existing ABL inhibitors. (2) the chronic lymphocytic leukemia demonstrated significant activation of PI3-kinase as compared to the normal B cells [56]. The only FDA approved combination product (idelalisib plus rituximab) for the treatment of leukemia were developed to inhibit not only the B lymphocytes but also the constitutively activated PI3-kinase [56,57], which therefore amplified the drug efficacy of this combination.

## Conclusion

In this study, FDA approved multi-target drugs and combination products targeting the human kinome were compared by the approach of network medicine. Popular targets of the multi-target drugs (VEGFR2 and c-Kit) and the combination products (EGFR and human DNA) were identified. As discussed, the approved multi-target drugs inhibited target pairs in the human kinome, while the combination products tended to target the much more diverse sets of target pairs. Moreover, similar mechanisms shared by approved multi-target drugs and combination products were identified by analyzing the sub-networks of 4 disease classes defined by the ICD. In sum, this study performed an analysis between approved multi-target drugs and combination products against the human kinome, which could help to identify novel target pairs efficacious for the next generation polypharmacology.

## Supporting Information

S1 Fig Sub-networks of drug-target interactions identified by disease classes treated by both multi-target drugs and combination products. Definition of hexagon, ellipse and round rectangle was the same as that in **Figs 4** and 5, and multi-target drugs were highlighted by an orange hexagon line.
(TIF)

S1 Table The established criterion of primary therapeutic targets. (PDF)

S2 Table A list of FDA approved multi-target drugs together with their corresponding approval indications and primary therapeutic targets. (PDF)

S3 Table A list of FDA approved combination products together with their corresponding approval indications and primary therapeutic targets. (PDF)

## Abbreviations

CDK: cyclin-dependent kinase
CLK: cdc2-like kinase
CML: chronic myelogenous leukemia
EGFR: epidermal growth factor receptor
FDA: U.S. Food and Drug Administration
GSK3: glycogen synthase kinase 3
HER2: receptor tyrosine-protein kinase erbB-2
ICD: International Classification of Diseases
JAK: janus kinase
MAPK: mitogen-activated protein kinase
MEK: mitogen-activated protein kinase kinase
PDGFR: platelet-derived growth factor receptor
PI3K: phosphoinositide 3-kinase
TK: tyrosine kinase
TKL: tyrosine kinase like
TNBC: triple negative breast cancer
TTD: Therapeutic Target Database
VEGFR2: vascular endothelial growth factor receptor 2
WHO: World Health Organization
