# Supplementary figures and images for: The Human Kinome Targeted by FDA Approved Multi-Target Drugs and Combination Products: A Comparative Study from the Drug-Target Interaction Network Perspective
Source: PLoS One. 2016 Nov 9;11(11):e0165737. doi: 10.1371/journal.pone.0165737 (PMC5102354; doi:10.1371/journal.pone.0165737)

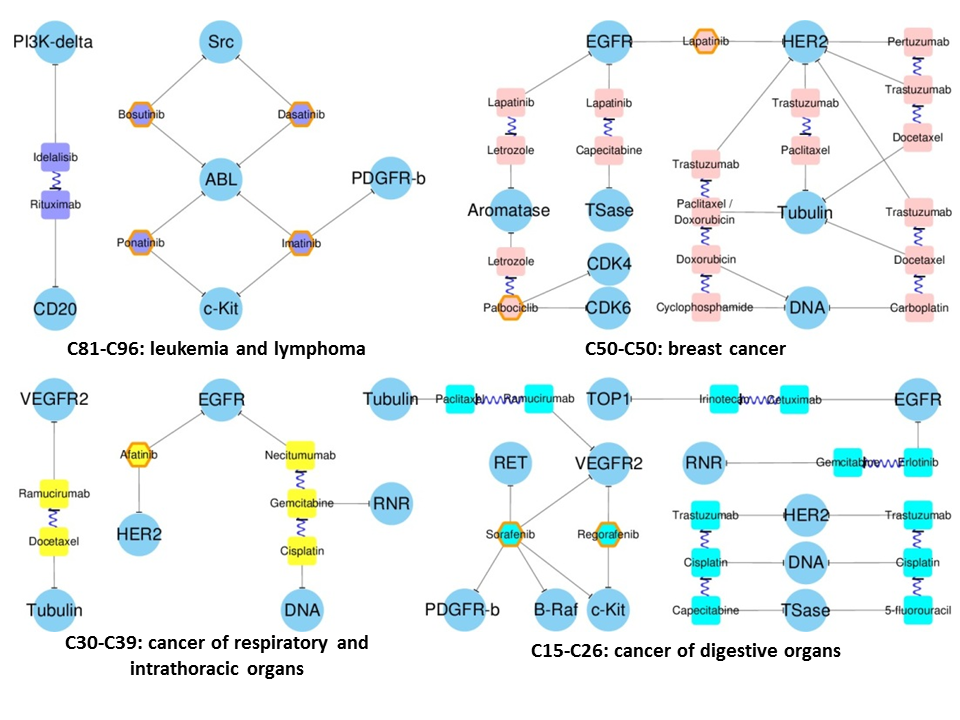

Supplement: S1 Fig — Definition of hexagon, ellipse and round rectangle was the same as that in Figs 4 and 5, and multi-target drugs were highlighted by an orange hexagon line. (TIF) [file pone.0165737.s001.tif]
