# Supplementary material for: The Human Kinome Targeted by FDA Approved Multi-Target Drugs and Combination Products: A Comparative Study from the Drug-Target Interaction Network Perspective
Source: PLoS One. 2016 Nov 9;11(11):e0165737. doi: 10.1371/journal.pone.0165737 (PMC5102354; doi:10.1371/journal.pone.0165737)
Supplement: S1 Table — (PDF) [file pone.0165737.s002.pdf]

S1 Table. The established criterion of primary therapeutic targets.

**Target**	A protein, DNA, RNA, cell wall/membrane component, or intra-cellular component unambiguously involved in the initiation or progression of a disease, and directly modulated by a drug with adequate potency. Potency criteria vary with assay, technology and target-type. Typically, drugs are expected to exhibit potencies of <500nM (ideally <100nM) in biochemical assays, but drugs of μM potencies may show adequate potencies in cell-based, in-vivo and clinical studies.
**Primary therapeutic target**	A target through which a drug mediates its claimed primary therapeutic effect, which is confirmed by biochemical assay and strong cell-based and/or in-vivo evidence linking the target to drug. Drug discovery against the same target is expected to lead to additional drugs of the same claimed therapeutic effect. Drugs typically exhibit potencies of <1μM in cell-based assays, but in some cases potencies of 10μM range may be acceptable. Criteria for in-vivo tests are less stringent.
**Secondary therapeutic target**	A target unambiguously involved in compensatory action or resistance against a drug (e.g. promoting alternative signaling or reducing drug bioavailability), and simultaneous action of a multi-target drug against this target and the main efficacy target exhibits statistically significant improvement of efficacy over that of the drugs against efficacy target only.
