# Supplementary material for: The Human Kinome Targeted by FDA Approved Multi-Target Drugs and Combination Products: A Comparative Study from the Drug-Target Interaction Network Perspective
Source: PLoS One. 2016 Nov 9;11(11):e0165737. doi: 10.1371/journal.pone.0165737 (PMC5102354; doi:10.1371/journal.pone.0165737)
Supplement: S2 Table — (PDF) [file pone.0165737.s003.pdf]

S2 Table. A list of FDA approved multi-target drugs together with their corresponding approval indications and primary therapeutic targets.

Drug Name	Code of ICD class	Disease indication	Target Name
Afatinib	C00-D49	Metastatic non-small cell lung cancer	EGFR; HER2
Bosutinib	C00-D49	Ph+ chronic myelogenous leukemia	ABL; Src
Cabozantinib	C00-D49	Metastatic medullary thyroid cancer	MET; VEGFR2
Dasatinib	C00-D49	Chronic myelogenous and acute lymphoblastic leukemia	ABL; Src
Imatinib	C00-D49	Ph+ chronic myelogenous leukemia	ABL; PDGFR-β; c-Kit
Lapatinib	C00-D49	HER2-positive metastatic breast cancer	EGFR; HER2
Lenvatinib	C00-D49	RAI-refractory differentiated thyroid cancer	VEGFR2; c-Kit
Nintedanib	J00-J99	Idiopathic pulmonary fibrosis	VEGFR2; PDGFR-α; PDGFR-β
Palbociclib	C00-D49	ER-positive and HER2-negative breast cancer	CDK4; CDK6
Pazopanib	C00-D49	Renal cell carcinoma and soft tissue sarcoma	VEGFR2; c-Kit; PDGFR-β
Ponatinib	C00-D49	Chronic myeloid and acute lymphoblastic leukemia	ABL; c-Kit
Regorafenib	C00-D49	Metastatic colorectal cancer and advanced gastrointestinal stromal tumor	VEGFR2; c-Kit
Sorafenib	C00-D49	Renal cell carcinoma and unresectable hepatocellular carcinomas	RET; VEGFR2; PDGFR-β; B-Raf; c-Kit
Sunitinib	C00-D49	Renal cell carcinoma and imatinib-resistant gastrointestinal stromal tumor	VEGFR2; c-Kit; PDGFR-β
Vandetanib	C00-D49	Medullary thyroid cancer	EGFR; VEGFR2
