# Supplementary material for: The Human Kinome Targeted by FDA Approved Multi-Target Drugs and Combination Products: A Comparative Study from the Drug-Target Interaction Network Perspective
Source: PLoS One. 2016 Nov 9;11(11):e0165737. doi: 10.1371/journal.pone.0165737 (PMC5102354; doi:10.1371/journal.pone.0165737)
Supplement: S3 Table — (PDF) [file pone.0165737.s004.pdf]

S3 Table. A list of FDA approved combination products together with their corresponding approval indications and primary therapeutic targets.

Drug Name	No. of Drugs	Code of ICD class	Disease indication	Target Name
Cobimetinib + Vemurafenib	2	C00-D49	Unresectable or metastatic melanoma	MEK (Cobimetinib)<br>B-Raf (Vemurafenib)
Dabrafenib + Trametinib	2	C00-D49	Unresectable or metastatic melanoma	B-Raf (Dabrafenib)<br>MEK (Trametinib)
Erlotinib + Gemcitabine	2	C00-D49	Advanced, unresectable or metastatic pancreatic cancer	EGFR (Erlotinib)<br>Ribonucleotide reductase (Gemcitabine)
Lapatinib + Capecitabine	2	C00-D49	Advanced or metastatic breast cancer	EGFR (Lapatinib)<br>Thymidylate synthase (Capecitabine)
Lapatinib + Letrozole	2	C00-D49	Hormone receptor positive metastatic breast cancer	EGFR (Lapatinib)<br>Aromatase (Letrozole)
Palbociclib + Letrozole	2	C00-D49	ER-positive,HER2-negative advanced breast cancer	CDK4, CDK6 (Palbociclib)<br>Aromatase (Letrozole)
Ramucirumab + Paclitaxel	2	C00-D49	Gastric or gastro-esophageal junction adenocarcinoma	VEGFR2 (Ramucirumab)<br>Tubulin (Paclitaxel)
Ramucirumab + Docetaxel	2	C00-D49	Metastatic non-small cell lung cancer	VEGFR2 (Ramucirumab)<br>Tubulin (Docetaxel)
Tofacitinib + Methotrexate	2	M00-M99	Moderately to severely active rheumatoid arthritis	JAK3 (Tofacitinib)<br>Dihydrofolate reductase (Methotrexate)
Trastuzumab + Paclitaxel	2	C00-D49	HER2-overexpressing metastatic breast cancer	HER2 (Trastuzumab)<br>Tubulin (Paclitaxel)
Idelalisib + Rituximab	2	C00-D49	Relapsed chronic lymphocytic leukemia	PI3K-delta (Idelalisib)<br>CD20 (Rituximab)
Cetuximab + Irinotecan	2	C00-D49	K-Ras wild-type, EGFR-expressing Colorectal Cancer	EGFR (Cetuximab)<br>Type I topoisomerase (Irinotecan)

Cetuximab + 5-fluorouracil	2	C00-D49	Squamous cell carcinoma of the head and neck	EGFR (Cetuximab)<br>Thymidylate synthase (5-fluorouracil)
Necitumumab + Gemcitabine + Cisplatin	3	C00-D49	Metastatic squamous non-small cell lung cancer	EGFR (Necitumumab)<br>Ribonucleotide reductase (Gemcitabine)<br>Human DNA (Cisplatin)
Pertuzumab + Trastuzumab + Docetaxel	3	C00-D49	HER2-positive metastatic breast cancer	HER2 (Pertuzumab)<br>HER2 (Trastuzumab)<br>Tubulin (Docetaxel)
Trastuzumab + Cisplatin + Capecitabine	3	C00-D49	HER2-positive metastatic gastric or gastroesophageal junction adenocarcinoma	HER2 (Trastuzumab)<br>Human DNA (Cisplatin)<br>Thymidylate synthase (Capecitabine)
Trastuzumab + Cisplatin + 5-fluorouracil	3	C00-D49	HER2-positive metastatic gastric or gastroesophageal junction adenocarcinoma	HER2 (Trastuzumab)<br>Human DNA (Cisplatin)<br>Thymidylate synthase (5-fluorouracil)
Trastuzumab + Docetaxel + Carboplatin	3	C00-D49	HER2-positive breast cancer	HER2 (Trastuzumab)<br>Tubulin (Docetaxel)<br>Human DNA (Carboplatin)
Trastuzumab + Doxorubicin + Cyclophosphamide + Paclitaxel	4	C00-D49	HER2-positive breast cancer	HER2 (Trastuzumab)<br>Human DNA (Doxorubicin)<br>Human DNA (Cyclophosphamide)<br>Tubulin (Paclitaxel)
Trastuzumab + Doxorubicin + Cyclophosphamide + Docetaxel	4	C00-D49	HER2-positive breast cancer	HER2 (Trastuzumab)<br>Human DNA (Doxorubicin)<br>Human DNA (Cyclophosphamide)<br>Tubulin (Docetaxel)
